# 骨髓增生异常肿瘤的骨髓微环境促进疾病发生发展的机制

**DOI:** 10.3760/cma.j.issn.0253-2727.2023.04.018

**Published:** 2023-04

**Authors:** 明华 洪, 春康 常

**Affiliations:** 上海交通大学附属第六人民医院血液科，上海 200233 Department of Hematology, Shanghai Jiao Tong University Affiliated Sixth People's Hospital, Shanghai 200233, China

骨髓增生异常肿瘤（myelodysplastic neoplasms，MDS）是一种以造血干细胞克隆增殖、骨髓增生异常、无效造血、外周血细胞减少，高风险发展为急性髓系白血病（AML）为特征的恶性疾病[1]。基因和表观遗传学变化及异常的骨髓微环境以多步骤和克隆进化的方式促成MDS或AML的转化[2]。但是，携带MDS常见基因突变的造血干祖细胞（hematopoietic stem and progenitor cells，HSPC）无独立的细胞自主增殖能力，并不能单独为恶性克隆提供增殖优势[3]。因此骨髓微环境的变化及其与HSPC的双向交流被认为在启动MDS的发生和支持肿瘤克隆的发展中起到关键作用。

在本综述中，我们主要关注MDS中骨髓微环境的异常及其促进疾病发生发展的可能机制，主要包括以下几个方面：①间充质干细胞（mesenchymal stem cells，MSC）如何促进MDS的发生发展；②骨髓中炎症与先天免疫信号异常的最新研究；③MDS-HSPC如何利用骨髓微环境获取克隆优势。

一、MSC如何促进MDS发生发展

MSC是骨髓造血微环境中最重要的细胞之一，可分化为脂肪细胞、骨细胞、软骨细胞以及其他胚胎谱系的细胞。MDS-MSC的克隆起源和存在的基因改变或染色体异常的意义尚不完全清楚[4]，MDS-MSC是原发的功能障碍还是MDS-HSPC诱发的次要影响，需要进一步思考。与健康的MSC相比，MDS-MSC表现出不同的表型，具有不同的突变、转录组和表观遗传学异常，突变负担更高，DNA复制压力增加，衰老和炎症基因表达水平升高[5]。

1. MSC对MDS的启动作用：MSC的某些原发性改变可以诱导骨髓发育不良并促成MDS或急性髓系白血病（AML）转化。MSC中β-catenin的激活性突变通过激活JAG1的表达，激活骨髓HSPC中的Notch 信号传导，从而改变HSPC的分化潜力，导致MDS或AML的发生[6]。另外，MDS的临床前模型，如Osx-GFP-Cre^+^ Dicer1^fl/fl^或SDS小鼠模型的建立同样证明了MSC对MDS的启动作用[7]。Zambetti等[8]通过对SDS小鼠模型和白血病前期患者的MSC的大规模RNA测序，确定了p53-S100A9-TLR4炎症信号轴在诱导HSPC DNA损伤的驱动作用。最近一项研究观察到在非造血系统中KRAS^G12D^的激活突变也可以导致类似MDS的表型，并且伴随着骨髓非造血细胞中多个炎症相关基因表达的上调，包括IL-1超家族成员和NLPR3炎症小体[9]，提示了MSC在启动MDS的发生中与炎症因子的重要联系。

2. MSC对MDS克隆的支持：与MDS-MSC的共移植，可以增强人MDS-HSPC在免疫缺陷小鼠中的植入[3]，这一现象直接提示了MSC对MDS克隆的支持。与健康MSC相比，MDS-MSC表现出异常的炎症信号增强了MDS-HSPC的功能[10]，因此炎症信号在MSC介导的MDS-HSPC支持中具有关键作用。另一方面，MDS-MSC维持健康HSPC正常功能的因子（如ANGPT1和KITL）的表达降低，同时重新编程健康的HSPC，使得HSPC的功能障碍持续存在[11]。需要说明的是，MSC启动和维持MDS的分子机制可能是重叠的，这些机制之间可能通过炎症或其他未知信号紧密联系。

3. MSC促进MDS疾病进展：MDS-MSC中WNT/β-catenin信号通路的激活可能导致AML的转化[6]。Li等[12]发现小鼠模型中β-catenin等位基因的缺失可以挽救del（5q）MDS无效的造血功能，并可能抑制疾病的进展。此外，MDS-HSPC和CXCL12^+^CD271^+^ MSC的直接接触，使HSPC暴露于高水平的CXCL12下，进而增加白血病细胞BCL-2的表达，促进白血病细胞的增殖[13]。最近几项研究表明了MSC的异常甲基化在促进疾病进展中的作用，但异常甲基化的关键位点仍需进一步探索。Bhagat等[14]发现WNT信号通路拮抗剂FRZB在MDS-MSC中的异常高甲基化和低表达导致WNT/β-catenin的激活。并且，由于异常甲基化导致MSC中SPINT2/HAI-2基因表达的下调有利于肿瘤干细胞的存活和黏附[15]。此外，Roux等[16]在MDS-MSC DNA甲基化谱的研究中观察到MSC的基因整体低甲基化并伴随DNMT1和UHRF1的表达降低，可能解释了部分MDS患者去甲基化药物治疗无效的原因。

二、炎症与先天性免疫失调

先天免疫和炎症信号的失调是MDS的重要标志。已有几篇综述对此作了非常详细的描述[17]–[18]。其中Toll样受体（Toll-like receptors，TLR）是先天免疫系统的重要组成成分，但TLR在不同危险度的MDS患者中的分布和意义尚不清楚。Paracatu等[19]指出TLR2和TLR4的信号传导与MDS中的细胞焦亡（pyroptosis）有关。其中TLR2及其伴侣TLR1、TLR6在MDS-HSPC中显著过表达，在低风险患者中导致HSPC的分化障碍和细胞死亡[20]。但TLR2也可能起到防止癌前细胞积累和最终转化的保护作用，因为在NUP98-HOXD13小鼠模型中，TLR2的缺失加速了白血病的转化[21]。最近在部分MDS患者中发现了TLR2上重现性的基因变异（TLR2-F217S）。在功能上，TLR2-F217S导致TLR2激动后的下游信号，如NF-κB和p38 MAPK的激活增强[22]。此外，免疫细胞上TLR的表达也影响了骨髓微环境。Li等[23]发现骨髓血管周围的树突状细胞表达高水平的TLR1/2，可引起骨髓微环境中成骨细胞活性和血窦内皮细胞数量的降低，同时，对TLR1/2的刺激可诱导IL-1β和其他炎性细胞因子在骨髓血管周围的分泌，进而调节HSPC的增殖。

TLR1/2/4以外其他类型的TLR在MDS中的贡献的研究较少。最近一项研究发现在内源性Rps14丢失的MDS中，通过激动TLR7与Myd88偶联，可以促进抗炎基因信号[24]，提示了TLR7在促炎通路和NF-κB通路之间的反馈调节。目前已经明确骨髓微环境中先天免疫和炎症信号传导失调在MDS发生发展中的重要性，但这些先天免疫和炎症信号因子随疾病进展的变化及调节机制仍不清楚，单个先天免疫和炎症信号因子对这一过程的确切贡献需要进一步研究和解决。

三、MDS-HSPC如何利用骨髓微环境获取克隆优势

慢性炎症是MDS-HSPC获得竞争优势的重要因素。与健康HSPC相比，MDS-HSPC中的先天免疫成分如TLR、TRAF6、IL8/CXCR2等信号通路过度激活[25]。其中，MDS-HSPC通过TLR-TRAF6介导的A20的激活，从经典的NF-κB信号转为非经典的NF-κB信号传导，来保护自身免受慢性炎症的损害，从而获得竞争优势[26]。而MDS-HSPC中IL-8/CXCR2自分泌回路的过度激活则通过加速细胞周期和增加下游致癌MAPK途径的信号传导，导致自身活力和克隆能力增强[27]。另外，HSPC携带的某些基因突变诱导了细胞对慢性炎症环境的耐受。Abegunde等[28]观察到TET2突变的HSPC在富含TNF-α的环境中具有克隆优势，同时它在响应炎症应激时增强了IL-6在内的炎症细胞因子的产生。IL-6诱导的Shp2-Stat3信号轴的过度激活，导致抗凋亡长链非编码RNA在TET2突变的髓系细胞中表达增加[29]，从而抵抗凋亡。

另一方面，富含TNF-α的炎症微环境可以通过NF-κB和pSTAT1/pSTAT3信号通路的激活诱导PD-L1在MDS-HSPC上的表达，而PD-L1^+^ MDS-HSPC具有内在的增殖优势并可诱导T细胞抑制[30]。此外，PD-L1介导的反向信号通过Akt/mTOR/HIF1α通路增强PD-L1^+^肿瘤细胞的糖酵解代谢，进一步促进肿瘤生长[31]。

因此，MDS-HSPC和健康HSPC应对MDS骨髓微环境成分变化的敏感性和调控机制的差异导致了这两类细胞不同的生存结局。关键的先天免疫和炎症通路改变了MDS-HSPC对炎症微环境的反应，并支持它们在慢性炎症环境中比健康HSPC更持续存在。

四、总结与思考

MDS独特的生物学特征，特别是复杂的突变结构和恶性克隆与骨髓微环境间的相互依存，阻碍了MDS临床前模型和药物开发的进展。近年来，科学家们在理解骨髓微环境对MDS发生发展的贡献方面取得了重大进展，加速了MDS临床前模型的建立，并提供了新的治疗靶点。炎症和先天免疫信号轴的几个组成部分正在被探索用于MDS的靶向治疗，尤其在低危MDS中[20]。而对于高危MDS患者，造血干细胞移植仍是治愈疾病的唯一方法，移植前疾病状态达到完全缓解有益于患者的生存。目前许多新药正在进行临床试验，其中多激酶抑制剂Rigosertib可以降低MDS-MSC的生存能力而不影响健康MSC，用于治疗去甲基化药物原发耐药的高危MDS患者[10],[20]。目前我们对大部分基因突变与骨髓微环境相互作用的机制知之甚少，后续对MDS遗传学、肿瘤干细胞和骨髓微环境的关联性研究有望进一步剖析MDS的发生发展机制，促进关键途径多靶点相结合的个性化治疗策略的开发。
